# Study protocol for the randomized controlled trial of EMBOLDEN: a multifaceted intervention aimed at Enhancing physical and community MoBility in OLDEr adults with health inequities using commuNity co-design

**DOI:** 10.3389/fpubh.2025.1555222

**Published:** 2025-04-24

**Authors:** Rebecca Ganann, Stuart M. Phillips, Sarah E. Neil-Sztramko, Kathryn Fisher, Elizabeth Alvarez, Ayse Kuspinar, K. Bruce Newbold, Caroline Moore, Maggie MacNeil, Heather Keller, Kylie Teggart, Lehana Thabane, Gina Agarwal, Diana Sherifali, Janet Adams, Aref Alshaikhahmed

**Affiliations:** ^1^Aging, Community and Health Research Unit, School of Nursing, McMaster University, Hamilton, ON, Canada; ^2^School of Nursing, McMaster University, Hamilton, ON, Canada; ^3^Department of Kinesiology, McMaster University, Hamilton, ON, Canada; ^4^Department of Health Research Methods, Evidence and Impact, McMaster University, Hamilton, ON, Canada; ^5^National Collaborating Centre for Methods and Tools, McMaster University, Hamilton, ON, Canada; ^6^School of Rehabilitation Science, McMaster University, Hamilton, ON, Canada; ^7^School of Earth, Environment and Society, McMaster University, Hamilton, ON, Canada; ^8^Department of Kinesiology and Health Sciences, University of Waterloo, Waterloo, ON, Canada; ^9^St. Joseph’s Healthcare – Hamilton, Hamilton, Canada; ^10^Department of Family Medicine, McMaster University, Hamilton, ON, Canada; ^11^Strategic Guiding Council, Resident of Hamilton, Hamilton, ON, Canada

**Keywords:** physical activity, nutrition, social participation, system navigation, health inequity, older adults, community-based intervention, public engagement

## Abstract

**Background:**

Reduced physical mobility is common in older adults and is associated with adverse outcomes, including functional decline, depression, social isolation, and poor nutritional status. Group-based programs focusing on physical activity and nutrition to support healthier lifestyles have demonstrated benefits, particularly when paired with social engagement activities. This paper presents the protocol for a randomized controlled trial (RCT) to test a lifestyle intervention called **EMBOLDEN**: a multifaceted intervention aimed at **E**nhancing physical and community **M**o**B**ility in **OLDE**r adults with health inequities using commu**N**ity co-design. EMBOLDEN is a co-designed 3-month intervention to improve quality of life by incorporating physical activity, healthy eating, social participation, and system navigation. Participants receiving the EMBOLDEN intervention plus usual care are expected to show improvement in physical activity and other health outcomes compared to receiving usual care alone.

**Methods:**

This is a 2-arm Type II hybrid effectiveness-implementation pragmatic RCT. Eligibility criteria include older adults (55+ years), community-dwelling in urban neighborhoods facing health inequities, able to speak or understand English or Mandarin (or access to family/friend interpreters), and able to walk 10 m unassisted by another person (assistive devices permitted). Participants will be randomized to the intervention or control arm (1:1 ratio). The intervention arm is usual care plus: (1) 12 weekly group-based sessions to increase knowledge/skills and behavior activation related to physical activity, healthy eating, fostering social connections and community resources; and (2) up to three tailored individual system navigation sessions. The control arm is usual care, in which participants identify and access services without research support. The primary outcome is time spent doing moderate-to-vigorous physical activity. Secondary outcomes will also be explored, including quality of life, life space mobility, depressive symptoms, nutritional risk, and loneliness. Data will be collected at baseline, 3 months (post-intervention) and 6 months. Mixed effects models will be used to analyze outcomes, intention-to-treat analysis will be employed, and multiple imputation will address missing data. Descriptive and qualitative data from participants, interventionists, and research documentation will be used to examine adaptations and implementation barriers/facilitators.

**Discussion:**

A community-based, co-designed lifestyle intervention may improve physical activity and other health outcomes in older adults living in neighborhoods with health inequities.

**Clinical trial registration:**

ClinicalTrials.gov, NCT05008159.

## 1 Introduction

### 1.1 Background and rationale

Mobility is necessary for maintaining independence and quality of life as we age. Mobility is defined as the ability to “move oneself within community environments that expand from one’s home to the neighborhood and to regions beyond” and is a holistic concept defined by five domains—physical, cognitive, psychosocial, financial, and environmental (1). This definition of mobility has been measured by various life-space mobility tools that comprehensively assess everyday movement, with studies showing that healthcare costs and utilization steadily increase across declining life-space scores (2). Reduced physical mobility is common with aging and a recognized precursor to frailty (3), a multidimensional syndrome of reserve loss (e.g., energy, physical ability, cognition) that leads to functional decline (4). Frailty also has bidirectional relationships with depression, loneliness, social isolation (5), and poor nutritional status, which may increase morbidity and mortality risk in older adult (6). Social frailty is a progressive state of being vulnerable to the loss or deprivation of resources necessary to fulfill one’s basic social needs throughout life; it is indicated by four factors: degree of neighborhood interaction, living alone, social participation, and economic status (7). Social frailty is receiving increased attention because these factors are potentially modifiable and because of its association with a higher incidence of adverse health outcomes including disability, reduced neuropsychological function, and mortality (7, 8).

Multi-faceted group-based interventions aimed at improving physical activity and nutritional behavior in older adults have demonstrated well-established health benefits (9–12); however, optimal design features of such interventions have been poorly defined. The inclusion of social support/engagement and other behavior change strategies in group-based nutrition interventions have been shown to further enhance their effectiveness (13). Community programs encouraging healthy, active lifestyles and social engagement may be highly effective in improving physical mobility, nutritional status, and social participation, older adults often face barriers to accessing these programs (e.g., social isolation, social anxiety, physical barriers, cost and transportation, lack of awareness of existing services) (14). These concerns are particularly notable for those living in communities with high health inequities (15–17).

Challenges arise when attempting to mobilize evidence or replicate—in the real world—the promising effects of home- or community-based mobility-enhancing interventions tested in controlled settings. For example, The LIFE study’s interventions (11) involved walking and lower extremity strengthening exercises in older adults aged 70–89 years and were mildly successful in reducing disability risk. Yet, their numerous exclusion criteria related to the presence of conditions common to older people (e.g., cancer, mental health, heart disease, diabetes, hypertension, arthritis) suggest that more modest results will likely be achieved if translated to the general population. The SPRINTT trial employs a multi-component intervention to prevent mobility disability but uses a controlled lab setting for the exercise component, has had limited testing to date in real-world settings (18, 19), and has not been fully assessed for its implementation and sustainability (20).

Ideally, interventions to enhance physical activity, social participation, and healthy eating should be designed to allow for tailoring to an individual’s lifestyle and personal preferences to capture a more realistic and pragmatic approach, avoid a ‘one-size-fits-all’ model, and facilitate implementation and sustainability. Engaging target populations and research partners to co-design such interventions may ensure better alignment between research and existing community programs, leverageable assets, and the contextualized experiences of older adults (21). Input from knowledge users (e.g., older adults, health and social service providers in the community, organizational decision-makers) can support mobilizing knowledge into practice to maximize impact.

Little research has explored effective and appropriately targeted interventions for physical and community mobility in older adults in a real-world context. This study aims to fill this research gap through a novel program co-designed with older adults in alignment with their needs. The research design will be outlined in the following sections and presented following the SPIRIT guidelines for clinical trial protocols (22).

### 1.2 Purpose and objectives

This protocol is for a randomized controlled trial (RCT) to test a novel intervention called **EMBOLDEN**: a multifaceted intervention aimed at **E**nhancing physical and community **M**o**B**ility in **OLDE**r adults with health inequities using commu**N**ity co-design. EMBOLDEN is a 3-month, co-designed, multifaceted, community-based primary prevention intervention incorporating physical activity, healthy eating, social participation, and system navigation. The primary objectives are to compare the effects between the intervention and control groups (usual care) on the primary outcome of physical mobility and to determine how the intervention was implemented and adapted across diverse urban neighborhoods. The secondary objectives are to compare the intervention and control groups on several secondary outcomes (e.g., life-space mobility, health-related quality of life, resilience, sense of community, nutrition status) and assess various implementation outcomes (e.g., adoption rate per protocol, intervention fidelity, feasibility and acceptability of the intervention to older adults and providers).

### 1.3 Trial design

The trial design is a 2-arm parallel group Type II hybrid effectiveness-implementation pragmatic RCT. The allocation ratio is 1:1 for the intervention and control arms. The hybrid design places equal emphasis on simultaneously assessing implementation and effectiveness (23). Including key implementation-related outcomes (e.g., uptake, contextual implementation determinants, and adaptations) is consistent with recommendations for developing and evaluating complex interventions; early consideration of implementation increases the potential to extract more effective implementation strategies, rapid translational gains, and contextually relevant information for decision-makers (24). The trial is likely to produce results similar to those expected in practice as a pragmatic design was chosen to more closely replicate real-world conditions. Design features, such as eligibility criteria, recruitment, and flexibility of delivery and adherence, were selected to best align with practice (25). The following pragmatic features were included in the EMBOLDEN trial design:

i) recruiting a sample with broad inclusion criteria reflective of the general population of community-dwelling older adults;
ii) conducting the study in existing community settings (e.g., community centers, recreation facilities);
iii) leveraging existing resources, staff, and service delivery organizations in the intervention arm that are currently available in the real world setting;
iv) supporting flexible delivery of the intervention (e.g., tailoring group-based strategies to participant group needs/interests/abilities and person-centerd tailoring of individual component), as would be done through other community programs in usual care;
v) allowing flexibility in adhering to the intervention in ways that are consistent with usual care;
vi) selecting primary and secondary outcomes that are participant-relevant (e.g., based on knowledge user input); and
vii) using intention to treat analyses based on all available data.

### 1.4 Research questions

The study is designed to answer several primary and secondary research questions. The primary research questions to be addressed are:

1 Effectiveness: Does the 3-month EMBOLDEN intervention plus usual care increase moderate to vigorous physical activity (MVPA) compared to usual care alone in community-dwelling older adults residing in areas of high health inequity?
2 Implementation: How is the EMBOLDEN intervention implemented and adapted across diverse urban neighborhoods?

The secondary research questions are:

1 Effectiveness:

i What is the effect at 3 months and 6 months of the EMBOLDEN intervention plus usual care compared to usual care on other outcomes (i.e., other smartwatch-derived measures of physical activity such as steps and distance traveled, self-reported physical activity, life-space mobility, nutritional risk, food security, health services utilization, health-related quality of life, resilience, loneliness, sense of community belonging, and collective efficacy) in community-dwelling older adults residing in areas of high health inequity?
ii If a significant treatment effect is observed for the primary outcome (MVPA), which subgroups of older adults benefit the most from the intervention?

2 Implementation:

i What is the adoption rate of the EMBOLDEN intervention (i.e., the proportion of participants enrolled in the study who attend at least one group session and receive at least one system navigation support session)?
ii What intervention dose is received by participants (i.e., number of group sessions and individual support sessions attended)?
iii Regarding recruitment:

a What is the recruitment rate for the trial, and how does the sample compare to the target population?
b What strategies contribute to the successful reach of older adults living in areas with health inequities?

iv How do local community organizations involved in delivering EMBOLDEN view the adoptability of the program within their organization?
v To what extent is the EMBOLDEN intervention implemented with fidelity, and what are the key implementation barriers and facilitators at individual, organizational, and community levels that impact fidelity?
vi How feasible and acceptable is the EMBOLDEN intervention to participants and interventionists?
vii How do participants and interventionists describe their experiences with EMBOLDEN, including satisfaction and perceived impacts of the intervention?
viii What factors are considered critical to the successful sustainment and scaling of the intervention?

### 1.5 Trial governance

The EMBOLDEN research team and a Strategic Guiding Council (SGC) will oversee the trial. It is anticipated that the SGC will include 26 members (18 service providers, 8 older adults). The SGC will engage older adult citizen partners and health and social service providers to co-design the intervention, guide implementation and refinements, and inform the evaluation and scalability assessment plan. The SGC will also be engaged in knowledge mobilization (e.g., developing key messages, presentations, peer-reviewed publications, lay-language summaries).

During the trial, the EMBOLDEN team will establish neighborhood-specific Community Advisory Boards (CAB), which will inform local adaptations and help address implementation challenges as they arise (e.g., recruitment, retention, interventionist-identified needs). The SGC will help to identify and recruit CAB members. CABs will comprise 6–8 older adult citizens and community providers (3–4 of each) within each identified neighborhood cluster (i.e., 2–3 neighborhoods geographically co-located). CAB members will provide input on necessary adaptations required to tailor to their local neighborhoods, be informed of trial outcomes as they are available, help interpret findings, and develop local knowledge translation strategies.

## 2 Methods and analysis

### 2.1 Participants, interventions, and outcomes

#### 2.1.1 Study setting

Participants will be recruited from 10 community settings in urban neighborhoods identified as areas of health inequity in the cities of Hamilton and Toronto in Ontario, Canada. The neighborhoods will be selected through a consultative process with SGC and CABs and informed by our completed environmental scan (26). Potential neighborhoods were identified in the scan using 2016 Census Tract (CT) data (27). CTs are small, relatively stable geographic areas that usually have a population of less than 10,000 persons (27). Potential neighborhoods were subsequently confirmed with 2021 census data and selected based on relatively higher proportions of their population with the following characteristics: age 55 and older, experiencing marginalization regarding material resources (composite score of education levels, those receiving government transfer payments, income, and unemployment rates) (28), immigrants, and older adults (aged 65 and older) living below the low-income cutoff. In Hamilton, eight neighborhoods were selected that had higher proportions of older adults and high levels of health inequity compared to the other neighborhoods across the city. In collaboration with an organizational partner in Toronto (Dixon Hall), two neighborhoods in their catchment were identified as having older adults members living in areas with health inequitites and fewer existing supports who could benefit from the intervention.

#### 2.1.2 Eligibility criteria

As a pragmatic trial, we chose broad inclusion criteria and will not exclude individuals based on existing chronic disease, comorbidities, or other factors that may impact attendance. All community-dwelling older adults (age 55 and older) living in a neighborhood selected for the study are eligible to participate in the study, with the only exclusion criteria being the inability to speak or understand English or Mandarin (at one site), the inability to walk 10 m without physical assistance from another person (assistive devices permitted), and lacking the capacity to consent to participate. Participants who do not speak or understand English or Mandarin (at one site) but have a family member/friend who can translate for them can participate with support.

#### 2.1.3 Intervention

The intervention is described following the TIDieR checklist (29).

##### 2.1.3.1 EMBOLDEN

**Theoretical underpinning**: The intervention and its core components are shaped by social cognitive theory targeting behavioral change through tailored and group-based approaches (30). An adapted experience-based co-design process with community-dwelling older adults and health and social service providers who were members of the SGC was used to refine and operationalize core intervention components (see below) (21). Qualitative research suggests co-design processes contribute to positive impacts at the participant, provider, and organizational levels (31). Our co-design process supported identifying key design features aligned with older adults’ priorities, delivery processes to match their preferences and ‘emotional touchpoints’ (i.e., maximizing positive experiences and addressing negative emotions that may be barriers to engagement). The co-design process used to engage the SGC has been evaluated. This included responses from a questionnaire, focus groups, and document analysis of meeting notes from 16 SGC meetings. Participants noted the strength of diversity within the SGC and engagement processes were perceived as inclusive and well-facilitated. The important impacts of the SGC in the preparation, execution and translational stages of EMBOLDEN were described by all participants, as well as the personal benefits of being involved as a member of the SGC (32).

**Core components:** The EMBOLDEN intervention will consist of usual care (see below) plus the following two core components: 12 weekly interactive group sessions and individually tailored 1:1 system navigation support. Both components will be delivered by the intervention team consisting of trained EMBOLDEN program facilitators who work in existing roles in community organizations and institutions (e.g., primary care interdisciplinary team members, municipal public health units, and municipal recreation departments).

As part of the program, we have engaged real-world providers who work for organizations that deliver similar types of programs, typically as one or occasionally two component(s) of the EMBOLDEN program. We offer the program in existing publicly funded facilities that operate within or nearby the neighborhoods (when a facility did not exist within the boundaries). We negotiated these partnerships and ensured alignment with their organizational mandates as part of the pragmatic trial to lay the foundation for sustainability.

Several adaptations to the intervention may be tailored to site-specific circumstances (e.g., language requirements and provider availability at partner organizations) while maintaining core intervention components; these adaptations are discussed below.

Core component #1—weekly group sessions: These group-based sessions will run each week for 12 sessions lasting approximately 120 min each. Group sessions will be delivered at an accessible, local community venue such as a recreation facility or community center located within or close to each priority neighborhood. Group sessions will focus on the following: (1) increasing knowledge, skills, and behaviors related to physical activity, healthy eating, and available community supports for older adults (including 30 min of facilitated physical activity delivered by a certified fitness instructor with experience and expertise working with older adults and interactive nutrition sessions led by the registered dietitian); (2) socialization activities to foster peer interactions and community connections, co-learning, and address social isolation; and (3) behavioral activation, goal setting, and skill-building to support independence and quality of life. The sessions will have a standardized and integrated curriculum to ensure core content of all curriculum threads is covered each week, with flexibility built into weekly topics and activities to allow the relative weighting and focus to be adapted to align with the interests and needs of a group (e.g., specific physical activities, interactive healthy eating activities, social activities, health promotion topics, community resources). Intervention participants will receive a Certificate of Completion at the end of the program.

Core component #2—system navigation: Up to three individually tailored, one-on-one sessions with a system navigator will be offered to participants virtually (via telephone or Zoom, according to participant preference). The sessions will be offered at the program’s beginning (~weeks 1–4 of group program), midpoint (~weeks 5–8) and end (~weeks 10–12). This facilitated system navigation support will be enabled by the Genie® system^1^ that we digitally mapped to each municipality’s local community information database (i.e., Red Book in Hamilton, 211 in Toronto). The Genie® system is an interactive, web-based self-management tool that maps participants’ social support network, elicits topics of interest (e.g., getting fitter, social activities, caregiving support), and supports individuals to connect with local programs and services, in alignment with their identified interests/priorities in a user-friendly “report.” During the encounter, the starts with gathering information about the participant’s demographics, including their postal code, to support later identifying resources located in geographic proximity to the participant. The facilitator then asks about the participant’s network that support them in their health and wellness goals, including informal and formal supports (e.g., health/social care providers), which acts as a visual aid to reinforce the person’s support network. The facilitator then gathers information about the participant’s interests and goals to provide them with resources, programs, and services in their community tailored to them. The facilitator enters participant’s data directly into the GENIE platform and can refer to the first encounter in subsequent sessions. The timing of the second and third individual system navigation sessions are flexible, according to participants’ expressed interests and needs. Participants will receive telephone or email system navigation session reminders, are free to opt out of these reminders, and will not be expected to reply to the messages unless they are unable to attend.

For any participants presenting with delirium or other emergent medical conditions during the study, the interdisciplinary intervention team would assess the situation and call for supports, if needed (e.g., family members, emergency responders).

**Interventionist training**: Intervention team members will receive 10–12 h of training on the curriculum (all components), facilitation skills, documentation (REDCap®), and system navigation software (Genie®). The intervention team is trained on ‘how to recognize signs of mental health distress’ and provided with relevant community resources and crisis supports to access, as needed. Through the orientation and training of these providers, we have strengthened capacity in the existing community-based workforce who work with older adults in these communities. While they are providing motivation and support within the EMBOLDEN program, they will continue to have these competencies when they return to their usual programming roles.

A training manual for delivering the EMBOLDEN program in English was developed and a manual to support delivery in Mandarin will be developed. Both will include detailed information for adaptations and tailoring all components to this linguistic and cultural group.

**Hamilton Mandarin site program adaptations:** One of the Hamilton sites will be comprised entirely of Mandarin-speaking older adults. The program will be modified in several ways to tailor the program to these participants, including language translation of all study documents and participant intervention materials, and delivery of all intervention components in Mandarin.

**Toronto sites program adaptations:** In response to limitations on resources and provider availability to deliver the system navigation component through an existing community service organization, lay community members experienced in connecting older adults with community resources will deliver this component and a physiotherapist working within a multi-service agency (versus primary care) was selected as the Program Coordinator at one site.

##### 2.1.3.2 Usual care

These services consist of older adults engaging in self-care and independently connecting with existing health and social service supports within the community to the extent that they are aware of them, self-initiate to access them, and overcome personal and system-based barriers without intervention or support by the research and/or intervention team. These services may include concomitant care/interventions, which are permitted in this trial due to its pragmatic nature.

#### 2.1.4 Pilot study

A pilot study was run to inform this trial. The primary aim was to determine the required sample size for this trial and to ensure that the recruitment procedures, data collection instruments and measures, and intervention components were feasible to deliver and acceptable to participants and providers. The pilot study was conducted over 9 weeks, delivered virtually (as it occurred during COVID and was subject to public health restrictions), and 10 participants were randomized (six intervention and four control, none involved in this trial). After finishing the pilot study, the participants were invited to give suggestions and feedback, which were incorporated into the trial design and this protocol. Pilot results informed the recruitment procedures (described below), confirmed that the intervention was feasible and acceptable to both participants and providers (with a strong preference for in-person delivery), helped determine the sample size for this trial, and resulted in the retention of all but one of the outcomes originally proposed (33).

#### 2.1.5 Outcomes

##### 2.1.5.1 Effectiveness

###### 2.1.5.1.1 Primary outcome

The primary outcome is time spent doing moderate-to-vigorous physical activity (MVPA) measured in minutes/day at each of the three timepoints (t_1_ = baseline, t_2_ = 3-months, t_3_ = 6-months). This outcome was selected because physical mobility was a priority in the EMBOLDEN intervention based on co-design, funding agency priorities, and high-level evidence cites it as one of the most common measures used to evaluate the long-term effectiveness of physical activity interventions (34).

Mean minutes/day of MVPA at each timepoint will be estimated using heart-rate data captured by a smartwatch (TicWatch Pro 2 Ultra GPS), using two heart-rate-based equations:

1 Maximum heart rate (MaxHR): MaxHR will be estimated using a formula appropriate for older adults [207-(0.7xAge)] (35, 36), and samples will be classified as light, moderate or vigorous based on the following %MaxHR: very light/light (<64%), moderate (64–76%), vigorous (>76%) (37). The time spent with a heart rate in the moderate or vigorous zone will be calculated for each wear day in order to calculate an average min/day of MVPA for each timepoint.
2 Heart rate reserve (HRR): also called the “Karvonen formula” (38, 39), this method uses %HRR to define physical activity intensity, where HRR = MaxHR – Resting HR (RestHR). MaxHR will be calculated as described above and RestHR will be calculated as the average of the daily minimum heart rate for 7 days. Samples will be classified as moderate/vigorous/light using the following formula: HRR × %Exertion, where moderate to vigorous intensity is % Exertion ≥40%. Samples in the moderate-vigorous categories will be aggregated and an average of minutes/day will be calculated for each timepoint as described above for MaxHR.

There is no clear guidance from the literature as to which approach is preferred, therefore, the change in the mean from baseline will be analyzed and reported for MVPA estimates from both algorithms.

###### 2.1.5.1.2 Secondary outcomes

GPS-derived mobility-related activities, including maximum distance traveled from home (km), daily step count (steps/day), and area of the convex hull [km^2^] (i.e., the minimum bounding geometry enclosing all the GPS loggings for each participant and the maximum area in which the participant engaged in activities), and GPS-derived quantity of out-of-home activities including number of trips (to out of home locations), homestay ratio (%), and time out of home (minutes) will be measured using smartwatch GPS data (40).

Secondary outcomes related to measure community mobility, self-reported physical activity, health and social service use, health behaviors, health status, quality of life, and community belonging will be collected via telephone interviews by trained research assistants using structured surveys. This includes self-reported health and social services utilization, including number of emergency department visits, number of hospitalizations, number of physician visits (general practitioner or specialist), number of visits with nursing or allied health professional (e.g., nurse, dietitian, pharmacist, physiotherapist, psychologist, counseling) and number of times community-based services used categorized by service (e.g., homemaker, meal service, adult day program) will be collected using the Health and Social Service Utilization Index (adapted for relevance to EMBOLDEN (41, 42)). We will also measure self-reported physical activity (Physical Activity Scale for the Elderly; PASE (43), community mobility (Life Space Assessment; LSA (44–46)), nutritional risk (Seniors in the Community: Risk Evaluation for Eating and Nutrition, Version II; SCREEN-II (47)), health-related quality of life (12-Item Short Form Survey; SF-12 (48) and EQ-5D-5L (49), resilience (Short Form 5-item Resilience Scale (50)), loneliness (6-item De Jong Gierveld Loneliness scale (51), physical activity literacy (Outcome Expectations for Exercise (OEE) scale (52, 53), depression (Patient Health Questionnaire-9; PHQ-9) (54), sense of community belonging, a measure of social capital at the neighborhood level (55), collective capacity within an individual’s social network (Collective Efficacy of Networks questionnaire; CENS (56)), food security (57), knowledge of chronic disease risk factors related to physical activity and healthy eating (Health and Behavior Assessment Tool; HABiT (58)), and food literacy (Short Food Literacy Questionnaire; SFLQ (59)).

These outcomes were selected recognizing the multi-faceted nature of the EMBOLDEN intervention and key areas it was intended to impact. Many of the above secondary outcomes have tools to capture the relevant data and guidelines for calculating total and/or subscale scores. The average completion time for secondary outcomes was 47.7 min (45–51) minutes based on pilot testing with older adult Strategic Guiding Council members (*n* = 3).

##### 2.1.5.2 Implementation outcomes

The selection of implementation outcomes was guided by the *Reach Effectiveness Adoption Implementation Maintenance (RE-AIM) framework* (60). This framework has been used to guide the planning and evaluation of health-related interventions from efficacy trials to implementation interventions (61). A definition of each item within the RE-AIM framework, along with the relevant EMBOLDEN measures, timing of data collection, and data sources, are provided in Table 1. Implementation outcomes for most framework domains include both quantitative and qualitative data. The “E” in the RE-AIM framework refers to effectiveness; thus, these outcomes (cited in Section 2.1.5.1 above) are also included in Table 1.

**Table 1:** Alignment of EMBOLDEN outcomes with RE-AIM framework.

RE-AIM outcome domain (58)	Measure	Data collection timepoint^a^	Data source (see data collection section for details)
R (Reach, Participant level)	-# Potential participants interested -# Eligible participants in the catchment area (by neighborhood using 2016 and 2021 census data)	t_1_	Recruitment Logs
	-# and % participants eligible (including demographics) -# Participants recruited by recruitment method	t_1_	Screening Logs
	-reasons for ineligibility	t_1_	Screening Logs
	-# and % participants enrolled (including demographics)	t_1_	Screening Logs
	-# and % participants retained (including demographics)	t_1_, t_2_, t_3_	Participant database
	-Reasons for dropout	t_1_, t_2_, t_3_	Exit survey
	-Representativeness of sample reached	t_3_	Census data
E (Effectiveness, Participant level)	-See Section 2.1.5.1 for a description of primary and secondary outcomes	t_1_, t_2_, t_3_	Participant Data Collection
	-Attendance—weekly group-based education sessions	t_2_	Group Session Attendance Logs
	-Completion of facilitated system navigation sessions	t_2_	System Navigation Attendance Logs
	-Satisfaction and perceived impacts - group-based sessions	t_3_	Mid and end of study survey (all intervention participants) Interviews (subset of intervention participants)
	-Satisfaction and perceived impacts – system navigation	t_3_	Interviews (subset of intervention participants)
	-Awareness of community programs/resources	t_3_	Interviews (subset of intervention participants)
	-Utilization of community programs/resources	t_1_, t_2_, t_3_ t_3_ up to 3 (start, mid- and end of group-based program)	End of study survey (all intervention participants) Interviews (subset of intervention participants) Genie® (intervention participants)
	-Social network analysis variables (e.g., size of the network, diversity of the network, number of strong/weak ties, number of hobby or community groups).	t_1_, t_2_, t_3_	Genie® (intervention participants)
Adoption (Organization level)	-# and % of community partners approached that join Strategic Guiding Council (SGC)	t_1_, t_2_, t_3_	SGC Recruitment Logs
	-Reasons for non-participation in SGC	t_1_, t_2_, t_3_	SGC Recruitment Logs
	-# and % of community partners retained in SGC	t_2_, t_3_	SGC Recruitment Logs
	-Reasons for discontinuation on SGC	t_2_, t_3_	SGC Recruitment Logs
	# and % of community partners approached and joined Community Advisory Board (CAB)	t_1_, t_2_, t_3_	CAB Recruitment Logs
	-Reasons for non-participation on CAB	t_1_, t_2_, t_3_	CAB Recruitment Logs
	-# and % of community partners retained in CAB	t_2_, t_3_	CAB Recruitment Logs
	-Reasons for discontinuation on CAB	t_2_, t_3_	CAB Recruitment Logs
	-# Community partners who agree to participate in intervention delivery among those approached	t_1_, t_2_, t_3_	CAB Recruitment Logs
	-# Trained interventionists within each neighborhood	t_1_, t_2_, t_3_	CAB Recruitment Logs
I (Implementation, organization level)	-Fidelity of delivery (% of core content delivered in group-based intervention sessions, content delivered during system navigation sessions)	t_2_	Group Session Checklist
	-Adaptations at local sites	t_1_, t_2_	Research Team Meetings with Interventionists (notes)
	-Barriers and facilitators to implementation	t_2_, t_3_	Research Team Meetings (notes) and Focus Groups with Interventionists (transcripts of audio-recordings) Interviews (intervention participants) CAB, SGC Meeting Notes
	-Cost of delivery (per participant, group, site)	t_1_, t_2_	Study Budget
M (Maintenance. Organization level)	-# Community partners intending to offer the program 3 months after study discontinuation^b^ ^−^Reasons for program cessation	t_3_	Key informant interviews

a t_1_ = baseline, beginning of study; t_2_ = 3 months, post-intervention; t_3_ = 6 months, end of study.

b The timeframe for assessing maintenance in RE-AIM is 6 months post-intervention. We adjusted the timeframe to 3 months to align with our final data collection timepoint (t_3_).

#### 2.1.6 Participant timeline

A number of activities will be conducted approximately 2 weeks prior to the beginning of the intervention (t_1_), including eligibility screening, informed consent, collection of demographic data, the first 7-day period for wearing the smartwatch, and randomization (performed after demographic data are collected and the participant has returned smartwatch after the first 7-day wear period). Time point t_1_ marks the beginning of the 3-month intervention period. Table 2 provides the details regarding the schedule for screening, wearing of the smartwatch, randomization, and assessments for participants. The intervention is designed for participants to start as a group and engage in the series of group-based sessions together.

**Table 2:** Timeline for EMBOLDEN participant screening, enrolment, and assessments.

Activity/assessment	Screening, informed consent, data collected prior to start of intervention t0	Baseline (start of intervention) t_1_	Follow-up post-intervention 3 months t_2_	Follow-up 6 months after baseline t_3_
Eligibility screen	X			
Informed consent	X			
Demographics, CaMos Frailty Index, GAQ, COVID-19 status^a^	X			
Randomization	X			
Smartwatch (worn 7-days *prior* to t_1,_ t_2_ and t_3_)	X		X	X
Primary Outcome: MVPA (minutes/day)		X	X	X
Secondary Outcomes: GPS-based mobility-related and out-of-home activities, PHQ9, LSA, Food Security, SCREEN II, SF-12, PASE, EQ-5D-5L, De Jong Gierveld, RS-5, CENS, Sense of Community Belonging, HABiT, OEE, SFLQ, Health and social services use^b^		X	X	X
EMBOLDEN program satisfaction survey			(also, mid-point or 1.5 months) X	

a Canadian Multicenter Osteoporosis Study (CaMos) collected to describe population at baseline (92), Get Active Questionnaire (GAQ).

b Patient Health Questionnaire-9 (PHQ9), Physical Activity Scale for the Elderly (PASE), Life Space Assessment (LSA), Seniors in the community: risk evaluation for eating and nutrition, Version II (SCREEN-II), 12-Item Short Form Survey (SF-12), Shortened Form 5-item Resilience Scale (RS-5), Collective Efficacy of Networks questionnaire (CENS), Health and Behavior Assessment (HABiT), Outcome Expectations for Exercise (OEE), Short Food Literacy Questionnaire (SFLQ), adapted Health and Social Services Utilization Index (HSSUI).

#### 2.1.7 Sample size

The results of the pilot study suggested a sample size of 250–500 for this trial (all sites) (33). The sample size calculation was based on the effect size for the primary outcome measure (MVPA) and appropriateness for the statistical model used to analyze the trial data (62). The following two-pronged approach was used to acknowledge the uncertainty in the pilot trial estimate of effect: (1) eliciting MVPA effect sizes from other relevant published studies, and (2) calculating the sample size for various plausible values of key parameters in the calculation using values from the pilot augmented by estimates from other published studies (63). A literature review identified two recent systematic reviews for interventions promoting physical activity: Wright et al. (64) and Larsen et al. (65). The review by Wright et al. (64) was most useful because of their comprehensive reach (included 2,762 trials), use of MVPA variance estimates based on survey data from 5 cycles (2007–2017) of the Canadian Health Measures Survey (*n* = 13,173 respondents age 18–79) (66); and breakdown of MVPA effect sizes by distributional anchors (e.g., 25th/50th/75th percentiles), different populations (e.g., older adults), and follow-up periods (e.g., 3+/6+/12+ months).

The following parameters were fixed in all sample size calculations: *α* = 0.05, two-sided hypotheses, power = 0.80, and attrition (90% retention at 3 months and 80% at 6 months as per pilot results). The *total* sample size ranged from 190 to 1,074, depending primarily on the assumptions regarding effect size and variance. Despite this wide range, the positive qualitative feedback from intervention participants suggested that the higher effect size seen in the pilot may be realized, thus leading to the final estimated range for the sample size of 250–500 (~50 per site).

#### 2.1.8 Recruitment and retention

Multiple methods will be employed to recruit and retain participants, with modalities, approaches, and messages that may attract a general older adult population based on existing literature and input from our SGC. Recruitment will begin by confirming the boundaries for the study neighborhoods since census tracts may not truly correspond to residents’ perceptions of their neighborhood. Engaging CABs who are well-informed about the local context will be instrumental to help the team develop effective recruitment and engagement strategies and local assets, understand and gaps/needs. In Hamilton census tracts the population size is typically 3,000–4,000 residents, with approximately 30% of the population over 55. Thus, the average neighborhood size to recruit from is 900 to 1,200 individuals. The two Toronto neighborhoods have 31,600 and 13,770 residents, with both having approximately 23% of the population over the age of 55, with 7,200 and 3,200 individuals available for recruitment, respectively.

Existing community organizational structures will be leveraged to attract and engage participants in each neighborhood using the following concurrent strategies:

1 **Doctors’ offices**: Family doctors will be approached to help with recruitment. Healthcare professionals are in a unique position to identify individuals in need as they tend to be a point of contact for people who might not otherwise be interacting with other individuals and/or community services and who could benefit from greater physical activity, support for healthier eating, system navigation, and social engagement (67). Encouragement from healthcare professionals can also help leverage involvement in a program. Various study materials will be provided to doctor/clinic offices, with their prior approval (e.g., PowerPoint slides for offices with TVs, posters for waiting areas and individual rooms, pamphlets/handouts for interested individuals to take home and review).
2 **Local communication channels**: Information on the study will be provided through established traditional media outlets (e.g., in local newspapers, community newsletters, radio, and television) and social media. While social media platforms may not directly reach all members of our older adult population, they may connect with agencies, family members and neighbors who could pass information on. All materials posted on communication channels will include a link to the flyer/poster for easy download/printing.
3 **Posters**: Study posters will be placed in community sites such as grocery stores, pharmacies, multi-unit buildings, community information sites, recreation centers, places of worship and seniors’ centers. Recruitment materials will be translated into different languages to foster inclusive programming as needed.
4 **Outreach services**: Older people who are socially isolated may be harder to recruit to the study. We will leverage existing services and supports to help reach this population, such as St Matthew’s House and Good Shepherd, two Hamilton-based community agencies providing support to older people experiencing food, housing or income security issues. These agencies also offer wellness programming and health system navigation. Staff may inform clients about EMBOLDEN and provide pamphlets when making porch visits/deliveries. Mail-ins to existing clients/members of community partner organizations by postal code is another strategy that may be employed.
5 **Voice platform**: This project will also be advertised on the Voice platform, a community engagement platform that McMaster University’s Institute for Research on Aging (MIRA) is licensing from the University of Newcastle. The platform offers a place where researchers can work with community members through community engagement opportunities and posted research studies (with ethics approval). Members of the public find out about Voice and posted research studies either through directly accessing the website, or through emails the platform sends out of new opportunities to registered users. The research team receives names and contact information of interested participants from the Voice platform, and the Research Coordinator follows up to confirm inclusion criteria and provide more details about the study.
6 **Direct mailings**: Directed mailings will be conducted if the above methods of recruitment do not secure enough participants. Direct delivery mailings through Canada Post to households in the targeted neighborhoods may be used. This recruitment strategy would be more costly and require a targeted approach so it will only be used if all other strategies are insufficient.^2^

All participants enrolled in the study, regardless of group allocation, will receive one $25 gift card for each quantitative data collection interview completed, for a maximum of three gift cards. This information is stated in all recruitment materials. Honoraria are provided to enhance recruitment by providing incentives to participate and mitigate attrition, particularly in the control group.

A cohort approach to recruitment and entry into the program will be adopted to foster socialization and relationship building (i.e., we aim to recruit all participants for a neighborhood to start within the first few weeks to allow intervention group participants opportunities to build relationships and socialize over the course of the program). One cohort will be rolled out per neighborhood with a maximum of 50 participants (25 intervention, 25 control) to be enrolled in each Hamilton (*n* = 8) and Toronto neighborhood (*n* = 2). Individual baseline data will be collected as individuals are enrolled in the study. The Research Coordinator will create monthly summary reports to enable the research team to determine whether recruitment targets are being met and whether additional targeted methods are needed to reach underserved groups. The final evaluation report will include the absolute number and percentage of individuals who responded to recruitment requests, enrolled, and retained in the program, as well as reasons for exclusion and dropout and comparison to the neighborhood characteristics collected through census data (see Table 1—RE-AIM outcomes and Table 2—Participant Timeline).

### 2.2 Assignment of interventions

#### 2.2.1 Allocation

The unit of randomization is the individual participant. The aim is to recruit 50 individuals in each neighborhood; these individuals will be randomized to receive the EMBOLDEN intervention versus usual care with a 1:1 ratio (25 individuals per trial arm at each site). The allocation process will use a randomized block design with three permuted block sizes (2, 4, 6). A biostatistician will generate the randomization sequence at each site using the *blockrand* package in R version 4.4.1.

#### 2.2.2 Allocation concealment and implementation

The random number sequences for each site will be input into a central web-based service (REDCap®) that allocates participants to the two study arms in accordance with the sequence. Once the randomization sequences for each site are set up in REDCap, the system will be moved to production mode, which locks the randomization model, thereby ensuring that the sequence is not modified once the project begins. A Research Assistant will screen participants for eligibility and obtain consent from those who accept an invitation to participate (see Supplementary material for consent form). Once informed consent is obtained, the participant will be enrolled, baseline data will be collected, and the Research Coordinator will then obtain the participant’s group allocation from REDCap.

#### 2.2.3 Blinding (masking)

Participants and interventionists will not be blinded. All data collectors will be independent of the intervention and blinded to treatment allocation. Statisticians will be blinded to treatment allocation.

### 2.3 Data collection, management and analysis

#### 2.3.1 Data collection methods

##### 2.3.1.1 Smartwatch data

The watches are distributed to study participants in both groups by priority postal mail and returned to the research team through the same mechanism. Study participants in both groups (intervention, control) will be instructed to wear the smartwatch for 7 days prior to baseline (t_1_), and for the first 7 days of t_2_ (3 months) and t_3_ (6 months). Since the smartwatch is intended to collect objective data on physical mobility rather than serve as part of the intervention to incentivize/motivate it, all watches will be configured to allow participants to view only the time and not their activity data.

The checklist by Matthews et al. (68) will be used to ensure our design, implementation, and reporting are consistent with objective standards for the use of physical activity devices in population-based research. Table 3 provides our design and implementation decisions relating to the items in their checklist. We are primarily interested in the following mobility-related measures: heart rate (sampling rate: 1 Hz), accelerometer data (sampling rate: 50 Hz), and GPS data (sampling rate: upon movement or 5-min intervals).

**Table 3:** Checklist for design, collection and reporting of physical activity monitoring data.

Feature/characteristic	Criteria
Device Selection	The TicWatch Pro 3 Ultra GPS was selected to capture mobility-related data from participants. The primary reason for selecting this smartwatch over others was the ability to access the raw data. Raw mobility-related data of primary interest includes heart rate (sampling rate: 1 Hz), accelerometer data (sampling rate: 50 Hz), and GPS data (sampling rate: upon movement or 5 min intervals).
Reliability and validity of the device	The TicWatch Pro 3 Ultra GPS device is a high-quality smartwatch; its activity-related measures offer the reliability and validity of other smartwatches in its class (e.g., Fitbit, Garmin, Apple). (93)
Wearing location (on body)	Participants will be instructed to wear the watch on their non-dominant hand during waking hours and to charge the watch nightly.
Target sampling period	Participants will be instructed to wear the watch for 7 consecutive days prior to t_1_ (baseline), and for the first 7-days of t_2_ (3-months) and t_3_ (6-months).
Participant tracking method (wear time)	Wear time each day will be determined based on the number of heart rate observations for the day; this is a more reliable indicator of wear time compared to start/stop times for daily heart rate samples as there can be gaps during the day where no heart rate data are captured (e.g., due to no/low battery, participant remove watch for bathing/swimming). Examination of the smartwatch data collected in the pilot study showed that heart rate data was captured consistently in accordance with the developer’s specified sample frequency (1 Hz).
Quality control checks	Heart rate data will be checked for validity (i.e., no negative or 0 readings), and invalid data will be removed prior to analysis. Duplicate samples (i.e., samples having an identical time stamp) will also be removed. Based on the examination of the smartwatch data from the pilot study, very few invalid samples were found (e.g., <0.1% of the data). Step count data will be checked for reasonableness (e.g., unreasonably high values for target population) and obvious errors (e.g., negative numbers for step counts indicating an invalid reading). The smartwatch captures cumulative steps for the entire wear time for the time point (seven days for participants the comply with the protocol). Ending step counts for a given day will be matched with starting step counts for the next day, and discrepancies will be identified and appropriately resolved. Based on the examination of the smartwatch data collected in the pilot study, step counts were reasonable with no obvious errors. GPS data are challenging to validate, especially for this target population, many of which live in apartment dwellings where a GPS signal is often obstructed, and satellite coverage can be variable/unreliable. The level of accuracy (in meters) will be determined based on the distribution of the values, density for the study area, needed accuracy to distinguish between locations etc. Invalid data will be removed from the dataset prior to analysis.
Compliance criteria	For step count and GPS-related measures, all days with at least 6+ hours of wear time (up to a maximum of 7 days/participant/time point) available data will be used in the calculations (i.e., if participants only have data for 5 of the 7 days for a given time point, these days will be used to generate measures, including averages, distance measures, trips).
Summary variables/categorizing data	1) Heart rate data will be used to calculate the primary outcome (MVPA in minutes/day). Heart rate samples will be categorized as vigorous/moderate/light using the algorithms described in Section 2.1.5.1. 2) Step count data will be used to calculate a secondary outcome measure related to mobility. The TicWatch captures the total step count for each day the watch is worn. The average daily step count for each time point (t_1_, t_2_, t_3_) will be calculated by averaging across the wear period for each time point (seven days for participants that comply with the protocol). 3) GPS data will be used to calculate a variety of distance-related measures (e.g., number of trips, maximum distance from home, homestay ratio). The algorithms used to generate the measures using GPS data will be implemented in R version 4.4.1 and based on the R Markdown guidance document provided by Muller and colleagues (94).

##### 2.3.1.2 Assessment tools data

Study data were collected and managed using REDCap electronic data capture tools hosted at McMaster University. REDCap (Research Electronic Data Capture) is a secure, web-based software platform designed to support data capture for research studies, providing (1) an intuitive interface for validated data capture; (2) audit trails for tracking data manipulation and export procedures; (3) automated export procedures for seamless data downloads to common statistical packages; and (4) procedures for data integration and interoperability with external sources.

Data will be directly entered into REDCap to track numbers of potential participants who contacted team based on interest in study, screened, eligible, recruited (eligible and provided informed consent). Data on weekly group-based program delivery fidelity and participant attendance will be captured by the Program Coordinator and entered into REDCap; Genie® data (e.g., social network, interests, community resources recommended) will be captured within Genie®, while data about participation in 1:1 sessions will be captured in REDCap. Mid- and end-of-program satisfaction surveys will be collected at the group program by the Research Coordinator as part of a site visit. These surveys are comprised of 5-point Likert-scale questions to explore overall impressions, satisfaction, and usefulness of program components/activities, followed by open-ended short text-answer questions to explore feedback on what contributed to their retention, perceived benefits/impact, barriers/challenges and future program enhancements.

Quantitative data will be collected from participants at each timepoint by independent research assistants blinded to allocation. Virtual data collection is expected to reduce participant burden and completion time, limiting the need to travel for both participants and research staff.

Qualitative data will be collected from a subset of intervention group participants using 1:1 semi-structured virtual interviews (by Zoom or telephone based on participant preference). Following the completion of each cohort, a subset of 5–6 participants per site will be identified to reflect a diversity of perspectives with respect to age, gender, socio-economic status, living status (alone or with others), and high vs. low engagement rates with program. Focus groups will also be conducted annually with the Hamilton intervention team to explore implementation determinants, adaptations and sustainability/scalability (with 1:1 interviews offered for those unable to attend a scheduled focus group). Separate focus group interviews will be conducted with the Mandarin-speaking and Toronto intervention teams. As part of the SGC and CAB meetings, questions will be posed to explore implementation, any adaptations necessary for successful research and program implementation in each neighborhood, as well as input into perceived sustainability and scalability at the final CAB meeting in each area; meeting notes will capture SGC and CAB input.

#### 2.3.2 Data management

All information will be kept confidential and study IDs will be used whenever data are coded. Records for this study will be in electronic (i.e., initial demographic and medical information, and questionnaires) and in paper forms. The electronic data from questionnaires will be housed on REDCap® through McMaster University network servers; the electronic Study Key (participant identification and contact information) will be stored in a file on McMaster University network servers and accessed through a password-protected computer. Once received, paper documents will be kept in a locked cabinet on campus. Only research team members will have access to data.

The Genie® System is provided as a hosted service and all data will sit on a server in Canada. We will use Microsoft (MS) Azure for hosting the data. The data will be backed up in Canada using MS Azure. MS Azure does not connect with any other services prior to Canadian servers. MS Azure’s encryption protocols erect barriers against unauthorized access to the data, including two or more independent encryption layers to protect against compromises of any one layer. MS Azure policies include the adoption of the international code of practice for cloud privacy, ISO-IEC 27018.

The smartwatch will be equipped to obtain GPS signals from the satellites to determine position (longitudinal and latitudinal coordinates). Only the coordinates for the destination information will be used to define parameters such as distance traveled. All GPS data will be encrypted with a secure password to prevent unwanted access in the case of loss of the device.

Users from the public register for Voice provide both their contact information and personal information (e.g., age, postal code, gender). The privacy and data storage policies have been reviewed and approved by the privacy and legal offices of both McMaster University and the University of Newcastle. Voice has rigorous guidelines in place to both protect data and ensure there is a rapid response in the unlikely event of a security breach. Data Security Protocols were reviewed and approved as part of the platform’s HiREB Application (#14929) and were also reviewed and approved by McMaster University’s Privacy Officer and Legal Office before the platform’s licensing agreement was signed.

#### 2.3.3 Analysis methods

##### 2.3.3.1 Baseline socio-demographic characteristics

Descriptive statistics will be used to compare treatment groups on baseline socio-demographic characteristics. Conventional statistical comparison of baseline characteristics of treatment groups (e.g., covariate imbalance *p* values) will not be reported; CONSORT 2010 guidelines discourage this in alignment with the widespread consensus that these methods are not appropriate to establish significant group differences in RCTs (69, 70).

##### 2.3.3.2 Effectiveness evaluation

Table 4 summarizes the outcomes/hypotheses/analyses pertaining to the primary, secondary, sensitivity and subgroup analyses. Treatment groups will be compared for the change from baseline for the primary and all secondary outcomes using a linear mixed model (LMM) or generalized LMM (GLMM). Mixed effects models are preferred in repeated measures clinical trials as these models handle missing data, more complex designs, and non-continuous outcomes (71). Generalized estimating equations (GEE) is another analytical option, although it is less often used due to its missing-completely-at-random (MCAR) assumption (72, 73). LMM/GLMM models generate estimates that are valid when the data are missing at random (MAR), which is recommended for the primary analysis in clinical trials as it is more reasonable than MCAR and less complex compared to missing-not-at-random (MNAR) modeling methods (74). GEE and LMM/GLMM all produce similar results if observed data are not related to missing data (73).

**Table 4:** Quantitative outcomes, hypotheses and methods of analyses.

Objective	Outcome, data type	Hypothesis^a^	Method of analysis^b^^,^^c^
Primary outcome analysis	Moderate-Vigorous Physical Activity (MVPA)	I, compared to C, shows increase in MVPA from baseline to 6 months	LMM/GLMM, REML estimation of model parameters, LSM (95% CI) for treatment group differences
Secondary outcome analysis	Life Space Assessment (LSA)	I, compared to C, shows increase in LSA score from baseline to 6 months	LMM/GLMM, REML estimation of model parameters, LSM (95% CI) for treatment group differences
	Short Form Health Survey, 12 Questions (SF-12): Physical Component Summary (PCS); Mental Component Summary (MCS)	I, compared to C, shows increase in PCS & MCS score from baseline to 6 months	LMM/GLMM, REML estimation of model parameters, LSM (95% CI) for treatment group differences
	EuroQoL 5 Dimension (EQ-5D): Today’s health; Mobility; Self-care; Usual activities; Anxiety/Depression; Pain	I, compared to C, shows increase in EQ-5D (today’s health, mobility, self-care, usual activities) and decrease in EQ-5D (pain, anxiety/depression) from baseline to 6 months	LMM/GLMM, REML estimation of model parameters, LSM (95% CI) for treatment group differences
	Patient Health Questionnaire (PHQ)	I, compared to C, shows decrease in PHQ score from baseline to 6 months	LMM/GLMM, REML estimation of model parameters, LSM (95% CI) for treatment group differences
	Physical Activity in Seniors (PASE)	I, compared to C, shows increase in PASE score from baseline to 6 months	LMM/GLMM, REML estimation of model parameters, LSM (95% CI) for treatment group differences
	Outcomes Expectation for Exercise (OEE)	I, compared to C, shows increase in OEE score from baseline to 6 months	LMM/GLMM, REML estimation of model parameters, LSM (95% CI) for treatment group differences
	De Jong Gierveld Loneliness Scale	I, compared to C, shows decrease in loneliness score from baseline to 6 months	LMM/GLMM, REML estimation of model parameters, LSM (95% CI) for treatment group differences
	Collective Efficacy of Networks (CENS)	I, compared to C, shows increase in CENS score from baseline to 6 months	LMM/GLMM, REML estimation of model parameters, LSM (95% CI) for treatment group differences
	Seniors in the Community Risk Evaluation for Eating and Nutrition (SCREEN II)	I, compared to C, shows decrease in SCREEN II score from baseline to 6 months	LMM, REML estimation of model parameters, LSM (95% CI) for treatment group differences
	Sense of community belonging	I, compared to C, shows increase in community belonging score from baseline to 6 months	LMM/GLMM, REML estimation of model parameters, LSM (95% CI) for treatment group differences
	Resilience	I, compared to C, shows increase in resilience score from baseline to 6 months	LMM/GLMM, REML estimation of model parameters, LSM (95% CI) for treatment group differences
	Short food literacy	I, compared to C, shows increase in food literacy score from baseline to 6 months	LMM/GLMM, REML estimation of model parameters, LSM (95% CI) for treatment group differences
	Food security: Worry will run out of food; Ran out of food	I, compared to C, shows decrease in food security concern from baseline to 6 months	LMM/GLMM, REML estimation of model parameters, LSM (95% CI) for treatment group differences
	Health and Behavior Assessment (HABiT): Concern (High Blood Pressure, Diabetes); Understanding (High Blood Pressure, Diabetes); Importance (Fruit/Veg, Salt, Physical Activity); Confidence (Improving Health Activities)	I, compared to C, shows decrease in concern and increase in understanding (high blood pressure/diabetes), importance (fruit/veg, salt, physical activity) and confidence (improving health activities) of concern baseline to 6 month	LMM/GLMM, REML estimation of model parameters, LSM (95% CI) for treatment group differences
Sensitivity analysis	Primary and secondary outcomes	Results from pooled analysis of multiple-imputed data sets will be consistent with original primary and secondary analyses	Explores impact of missing data using multiple imputation by chained equations (MICE), predictive mean matching, imputation by treatment group; employs modeling approach used in main analyses (i.e., LMM/GLMM, REML, LSM & 95% CIs)
Subgroup analysis	Moderate-Vigorous Physical Activity (MVPA) by sex category (male, female, other)	Treatment effect will be consistent across sex categories	Performed if overall treatment effect observed; employs modeling approach used in main analyses (i.e., LMM/GLMM, REML, LSM & 95% CI); separate models run for each category, interactions test (treatment group × sex) provided
	Moderate-Vigorous Physical Activity (MVPA) by 10-year age categories	Treatment effect will be consistent across age categories	Performed if overall treatment effect observed; employs modeling approach used in main analysis (i.e., LMM, REML, LSM & 95% CI); separate models run for each category, interactions test (treatment group × age) provided

a I = Intervention Group, C = Control Group.

b LMM = linear mixed model, GLMM = generalized linear mixed model, REML = restricted maximum likelihood estimation, LSM = least squares means (=model predicted means), CI = confidence interval.

c LMM/GLMM models will include the following fixed effects: outcome value at baseline, time (categorical), treatment group (categorical), and treatment × time interaction (categorical). Random effects for participants and sites will include either random intercepts, or the more complex model (random intercepts and random slopes) if suggested by spaghetti plots and fit statistics.

The dependent variable in the LMM/GLMM models will be the observed outcome value at each scheduled follow-up point (t_2_ = 3 months, t_3_ = 6 months). Independent variables in the model will include the following fixed effects: baseline outcome value, categorical factors for the treatment group (intervention, control), time (t_2_ = 3 months, t_3_ = 6 months), and time × treatment group interaction. Models that analyze change scores versus outcome scores, both adjusted for baseline, yield the same results (72). The model will also include random effects for sites and participants PRINTDATE \* MERGEFORMAT 2/17/25 4:28:00 PM either the random intercepts model or the more complex random coefficients (random intercepts and slopes) model, based on examining spaghetti plots and comparing fit statistics for the two models (e.g., AIC, BIC). Ten sites are considered sufficient for exploring these random effects (75). Although we do not expect significant gains with the more complex models due to the homogeneous nature of our trial sites (all health inequity sites within two close-proximity urban cities), the more complex random coefficients model will be retained if spaghetti plots suggest that participants and/or sites are responding differently over time, and the fit statistics indicate significant gains resulting from the extra model complexity. Parameters will be estimated using restricted maximum likelihood estimation (REML) and the Kenward-Roger method for calculating the degrees of freedom. Differences between least squares will estimate treatment group comparisons for each outcome at each time means (LSMs = model predicted means), with accompanying *p*-values and 95% confidence intervals.

Models will not be adjusted for baseline covariate imbalances. Adjustment for baseline variables is recommended when there are anticipated strong associations between baseline characteristics and the primary outcome (76, 77). We are not aware of strong prognostic baseline characteristics affecting the primary outcome and site selection employed homogenous health inequity criteria; under these conditions, baseline imbalances are most likely attributable to chance (not information/selection bias); thus, unadjusted analyses are expected to provide unbiased estimates of treatment effects (77).

A sensitivity analysis will be performed to further explore the impact of missing data. Multiple imputation will be employed with an imputation model that includes ancillary variables thought to be predictive of the probability of missingness and/or predictive of the responses. Multiple imputation by chained equations (MICE) will be conducted, with predictive mean matching used to avoid invalid values and ensure robustness regarding the misspecification of the imputation model (78). Although the analysis model will be simpler than the imputation model, this incompatibility is not a major concern (72). The number of imputations will be determined using the rule of thumb based on the percentage of participants with missing data (e.g., 70 imputed data sets if 70% of participants had any missing data) (79). The statistical model used in the primary analysis (LLM/GLMM) will be applied to the multiple data sets, and the results will be pooled using Rubin’s rules (80). For greater robustness, we will adopt the recommendation to conduct the multiple imputation separately by randomized group (81).

If a significant treatment effect is observed for the primary outcome (MVPA), a subgroup analysis for the primary outcome will be conducted in accordance with the following three rules: (1) the analyses should be regarded as secondary and exploratory, (2) the number of subgroups should be restricted to a select few, and (3) the subgroups should be specified *a priori* (82). The subgroups analysis will involve the following categorical variables: sex/gender (male/female/other) and age (10 year age categories). Subgroup analyses will also be performed on the imputed datasets, with imputations done separately by a randomized group as recommended (81).

##### 2.3.3.3 Qualitative data analysis

Qualitative descriptive methodology (83, 84) will guide the exploration of participant experiences, and a hybrid conventional (inductive) and directed (deductive) content analysis approach will be used in analyzing the data (85). Data collection and analysis will be iterative; issues arising in early interviews will be explored further in subsequent interviews. Transcripts and meeting notes will be analyzed independently by two research team members using NVivo software (Lumivero) with a constant comparative method in which new information is compared to previous information (86). The initial coding tree will be reviewed with the broader qualitative team to establish broader categories and ensure consistent approaches across coders. Any coding discrepancies will be resolved by discussion. The data will be interpreted with input from the SGC.

##### 2.3.3.4 Implementation evaluation

The implementation evaluation is designed as a mixed-methods formative evaluation to allow for iterative refinement of implementation strategies and to inform future scale-up and scale-out. Implementation evaluation will be guided by the RE-AIM framework (60). Table 1 provides the outcomes to be measured corresponding to each of the RE-AIM items. We will use quantitative data to examine implementation outcomes and qualitative data to explore the process of implementation.

The primary implementation outcomes will be analyzed using descriptive statistics expressed as percent/rate and corresponding 95% confidence intervals.

The process of implementation will be evaluated by examining qualitative data from several sources (committee documents/notes and meetings with interventionists, interventionist interviews, participant experience surveys, semi-structured interviews with SGC members reflecting diverse roles, focus groups with CAB members, and data sources both during and after the intervention. The data will be coded and analyzed using NVivo software, using the updated Consolidated Framework of Implementation Research (CFIR) (87) as a preliminary coding tree; codes will be revised using inductive analysis to identify data that do not align with the CFIR framework or to elucidate nuances within the CFIR domains and constructs. We will explore implementation determinants from the perspective of participants and interventionists. NVivo matrix queries will be used to explore differences in implementation determinants by perspective and site.

##### 2.3.3.5 Community and citizen engagement evaluation

The community and citizen engagement strategy embedded throughout the EMBOLDEN research program, inclusive of this trial, will be evaluated annually by members of the SGC using the Patient-Centered Outcomes Research Institute’s Ways of Engaging-Engagement Activity (WE-ENACT) and Engagement Activity Inventory (NET-ENACT) Tools (88, 89).

##### 2.3.3.6 Social network analysis (intervention participants only)

Network characteristics data will be extracted from the system navigation intervention tool, Genie®, at up to three timepoints depending on the uptake of this intervention component. We will construct network measures such as: (1) size of network (number of network members on the diagram), (2) diversity of network (number of types of relationships on the diagram, e.g., close family, distant family, acquaintances, friends = 4; close family = 1), (3) number of weak ties A (not family or friends; e.g., acquaintances, neighbors, colleagues), (4) number of weak ties B (number of network members in the outer circle of the diagram), (5) number of hobby or community groups, (6) frequency of contact (a sum, based on the frequency of contact with all network members, 4 = every day, 3 = at least once a week, 2 = at least once a month, 1 = less often), (7) number of women in the network, (8) number of pets. These will be used to develop measures of network change from Genie® visit 1 to Genie® visits 2 and 3 (as available). The questions we will explore in the network analysis include:

- What is the network structure of older adults with health inequalities?
- What are the differences in network structure and network change for older adults living with or without a partner and of different socio-economic status?
- What are the predictors of positive network change for older adults with health inequalities?
- Is there an association between network structure and the support, health and well-being of older adults with health inequalities (e.g., change in physical activity; loneliness, health-related quality of life, social support)?

Genie® network data will be entered into statistical software using de-identified data by a member of the EMBOLDEN team. The network data for each participant will then be added to data collected in the intervention arm of the trial and only the anonymized data will be analyzed to answer the above questions. All data will be analyzed descriptively.

### 2.4 Monitoring

#### 2.4.1 Governance structure

The EMBOLDEN research program, inclusive of this trial, is overseen by the interdisciplinary research team and a city-wide Strategic Guiding Council (SGC). The trial will also be informed by Community Advisory Boards (CABs). See Section 1.5 for the role of the SGC and CABs.

#### 2.4.2 Data monitoring committee

A data monitoring committee (DMC) is not required for this trial as the risk of severe adverse events is low. This trial will not compare rates of mortality or morbidity, nor will this trial administer an invasive treatment. In addition, the brevity of this trial (3-month intervention) does not lend itself to a DMC (90). In lieu of a formal committee, the implementation of the trial will be reviewed on a regular basis by members of the research team, the SGC, and local CABs.

#### 2.4.3 Safety/harms

Although there is minimal risk of entering this study, there are some risks to starting or increasing any physical activity, such as injury, fatigue, fainting, abnormal blood pressure, irregular heart rhythm, and, in very rare instances, heart attack or death. The benefits of increasing mobility outweigh most of the risks of potential injury, and having trained team members and setting realistic goals will allow for gradual adjustments in participants’ mobility levels. Every effort will be made to minimize these potential risks by evaluating preliminary information relating to an individual’s health and fitness, and a trained and certified facilitator will provide adaptations to each exercise. Participants will be required to complete the Get Active Questionnaire (GAQ) developed by the Canadian Society for Exercise Physiology (CSEP) to participate in the trial. Any responses of concern to the GAQ will be reviewed by a staff member from the Physical Activity Centre of Excellence (PACE, led by a Co-Principal Investigator on this study). PACE staff are responsible for providing exercise programming and adapting the exercise prescription appropriately to older adults and individuals with several high-risk health conditions, including multiple sclerosis and spinal cord injury, those undergoing active cancer treatment, and cardiac rehabilitation.

We also aim to mitigate these potential risks by reiterating the declaration form at the beginning of each exercise class. Additionally, the session moderator will monitor and make careful observations of the participants during each group session. To ensure safety of the participants during this study, individuals will be asked to report any adverse symptoms to the Program Coordinator at the end of each session; we will also ensure that the participant has provided us with their home address and an emergency contact. If an abnormal event occurs and/or health is compromised, the Coordinator can provide further guidance to the participants, alert the emergency contact, and contact emergency services if required.

The PHQ-9 will be used to manage risk related to symptoms of depression. The clinical research assistant will review the PHQ-9, in particular question 9, which indicates thoughts of harming oneself and consult with the clinician on the study team if necessary. There may also be risks involved, including anxiety or fatigue, when completing the study questionnaires and interviews. Participants can refuse to answer any questions or stop the interview at any time without consequence. Several Co-PIs are healthcare professionals and have oversight of the research assistants interviewing participants (many of whom are regulated health professionals themselves) and administering intervention/control conditions and are qualified to monitor participants to assess any risk. All participants, regardless of assigned condition, will continue to have access to their usual care. If participants are seen in distress, fatigued, or in anxious situations, the trained research assistants will attend to the participant’s needs and seek out the proper resources and assistance if necessary.

This study will use Zoom for Healthcare to collect data, which are externally hosted cloud-based services. While the Hamilton Integrated Research Ethics Board has approved using the platforms to collect data for this study, there is a small risk of a privacy breach for data collected on external servers. Zoom for Healthcare is compliant with healthcare security measures in accordance with the Canadian Personal Information Protection and Electronic Documents Act (PIPEDA).

### 2.5 Ethics and dissemination

#### 2.5.1 Research ethics approval

Prior to the commencement of the study at the Hamilton sites, the study protocol was submitted to and approved by the Hamilton Integrated Research Ethics Board (HiREB), Hamilton, Ontario, Canada (HiREB 2021-13387-GRA). Research ethics approval has been secured through the Toronto Public Health Research Ethics Board and is currently being sought from Unity Health Toronto before initiating the study at the Toronto sites.

#### 2.5.2 Protocol amendments

Research ethics boards will be notified of any protocol modifications, and changes will be made based on their recommendations. A log of protocol amendments will be kept and made available to all relevant parties. At the time of writing, a number of protocol amendments for various proposed changes have already been submitted and approved by HiREB (e.g., replaced original resilience measure with a shorter validated scale, increased honoraria to reflect receipt of additional funding, added a number of outcomes/assessments required by funding agencies, updated sample size and sites to reflect additional funding, shifted to in-person delivery to reflect the lifting of COVID-related restrictions, combined recruitment sites to increase the likelihood of reaching the target sample for the neighborhood). The study protocol presented here is in accordance with the most recent version of the protocol approved by HiREB (version 13; April 15, 2024).

#### 2.5.3 Consent

A research assistant will explain the nature of the study, their rights as study participants, confidentiality of their data, voluntary entry into the study, and their ability to withdraw from the study at any time. Any questions will be answered, and informed consent will be obtained prior to enrolling any participant into the study.

After the participant has reviewed the consent form in detail, the research assistant will explain that they are going to ask a few brief questions about the study (Brief Assessment of Capacity to Consent (BACC) (91). The BACC is an 11-item scale that typically takes less than 5 min to administer. Potential participants will have a copy of the consent form in front of them to during the consent process and do not have to rely solely on their ability to memorize the protocol details when given consent to enroll. The research assistant will revisit, review, and re-educate for reinforcement and ask the question again as necessary. Adequate capacity to consent is determined by a score of 8 or higher (out of a possible 11).

#### 2.5.4 Confidentiality

All study personnel will be trained and monitored regularly in the requirement of participants’ confidentiality according to research ethics board regulations and following good clinical practice guidelines. All research-related procedures, including data collection and storage, will be carried out on McMaster University’s network, a secure server. No information about any participants will be shared outside of the research team without prior consent unless there are concerns regarding participant health and safety, and in this instance, these concerns will be communicated to the participant and appropriate healthcare professionals.

Voice has rigorous guidelines in place to both protect data and ensure there is a rapid response in the unlikely event of a security breach. To date, Voice has not experienced a security breach. Voice is well-supported by an expert IT and user design team based out of the University of Newcastle that prioritizes the security and protection of user data. Data Security Protocols were reviewed and approved as part of the platform’s HiREB Application (HiREB #14929) and were also reviewed and approved by McMaster University’s Privacy Officer and Legal Office before the platform’s licensing agreement was signed.

#### 2.5.5 Declaration of interests

No investigators, members of the research team, and/or their partners or immediate family members will function as an advisor, employee, officer, director or consultant for a study-related sponsor or funding source; have a direct or indirect financial interest in the intervention employed in this research study; nor receive any personal benefit (apart from fees for service) as a result of, or connected to this study (e.g., remuneration, intellectual property rights, rights of employment, consultancies, board membership, share ownership, stock options, honorariums).

#### 2.5.6 Access to data

Only authorized research personnel can access the data. The study key will be destroyed once data collection is completed, cleaning of the data is completed, and the researchers no longer need access to identifiable information.

#### 2.5.7 Ancillary and post-trial care

Given the nature of the EMBOLDEN intervention and its target population, no ancillary or post-trial care is envisioned. No harms are anticipated from participation in the trial; thus, no compensation is required.

#### 2.5.8 Dissemination strategy

We plan to present at academic conferences and publish peer-reviewed journal articles on the innovative study methodology, lessons learned through the collaborative approach to research implementation, and findings from both implementation and effectiveness evaluations. All research team members that have made meaningful contributions to the content of manuscripts will be invited as co-authors, and required to review and approve all manuscripts prior to publication. Knowledge translation strategies will feature community input with respect to content, format, and dissemination strategies. We will work in collaboration our community partners to co-produce dissemination materials (e.g., lay-friendly research summaries, briefs, infographics) in addition to traditional academic papers and presentations.

## Footnotes

^1^ https://www.genie.soton.ac.uk/eng/

^2^Snap Admail https://www.canadapost-postescanada.ca/sam/.
